# Advances in Cytotoxicity Testing: From In Vitro Assays to In Silico Models

**DOI:** 10.3390/ijms262211202

**Published:** 2025-11-19

**Authors:** Barbara Ziemba

**Affiliations:** Department of General Biophysics, Faculty of Biology and Environmental Protection, University of Lodz, 141/143 Pomorska St., 90-236 Lodz, Poland; barbara.ziemba@biol.uni.lodz.pl; Tel.: +48-42-635-41-44

**Keywords:** cytotoxicity assays, toxicity testing, 3D cell culture, organ-on-a-chip, high-content imaging, in silico models, integrated testing strategies (IATA)

## Abstract

Cytotoxicity testing remains a cornerstone of modern toxicology, providing critical insight into how chemicals and drugs affect cell viability and function. Classical colorimetric assays such as MTT, LDH release, and neutral red uptake established the methodological basis of in vitro toxicology and continue to serve as regulatory benchmarks. However, their limited mechanistic depth and physiological relevance have prompted the field to evolve towards more predictive and human-centred approaches. Recent advances in high-content imaging, flow cytometry, and real-time impedance analysis have transformed cytotoxicity testing into a multiparametric discipline capable of detecting adaptive and sub-lethal cellular responses. Parallel progress in computational toxicology has introduced in silico models—QSAR, machine learning, and physiologically based pharmacokinetic (PBPK) modelling—that enable quantitative in vitro–in vivo extrapolation (QIVIVE). The integration of these computational tools with 3D organoids, organ-on-chip systems, and stem cell-based models allows for cross-validation between predictive simulations and experimental evidence, enhancing mechanistic interpretation and translational accuracy. Together, these developments underpin New Approach Methodologies (NAMs) and Integrated Approaches to Testing and Assessment (IATA), marking the transition from descriptive assays to predictive, mechanism-anchored frameworks that bridge in silico prediction with in vitro and in vivo validation—advancing both biomedical research and regulatory toxicology.

## 1. Introduction

Cytotoxicity testing remains a central pillar of modern toxicology, linking cellular responses to hazard identification and risk evaluation in biomedical research, pharmaceutical development, and chemical safety assessment [1,2,3]. By quantifying changes in cell viability and function, these assays provide critical data for understanding toxic mechanisms and supporting regulatory decisions [4,5]. Agencies such as the United States Food and Drug Administration (FDA), the European Medicines Agency (EMA), and the Organisation for Economic Co-operation and Development (OECD) require cytotoxicity data before compounds advance to preclinical or clinical stages [3,5,6]. In parallel, the ethical principles of the 3Rs—Replacement, Reduction, and Refinement—have accelerated the adoption of non-animal testing strategies [1,2]. The influential report Toxicity Testing in the 21st Century further highlighted the need for human-relevant, mechanism-based approaches [7].

Historically, cytotoxicity was evaluated using two-dimensional (2D) cell cultures combined with simple colorimetric or fluorometric readouts. The MTT assay, which measures mitochondrial reduction in tetrazolium salts, became a long-standing standard [8,9]. Complementary methods such as lactate dehydrogenase (LDH) release, reflecting plasma membrane integrity [10], and neutral red uptake (NRU), which probes lysosomal activity [10,11], provided alternative functional perspectives. Although inexpensive and reproducible, these classical assays offer only limited mechanistic insight and show variable correlation with in vivo outcomes [4,12]. Nonetheless, they established the methodological foundation of in vitro toxicology and remain useful as reference points in regulatory practice [3,5].

As toxicology advanced, the limitations of single-endpoint assays became increasingly evident. High false-positive rates in classical genotoxicity tests—especially in p53-deficient rodent cell lines—emphasised the need for more predictive, human-based models [13]. The emergence of large-scale high-throughput initiatives such as Tox21 [14] and ToxCast [15] enabled systematic profiling of thousands of compounds across multiple cellular pathways [16]. At the same time, stem cell-derived systems, including human embryonic stem cells (hESCs) and induced pluripotent stem cells (hiPSCs), expanded the ability to study developmental and organ-specific toxicity in human-relevant settings [17,18]. The rapid growth of nanotechnology introduced new challenges and gave rise to nanotoxicology—a distinct field requiring dedicated in vitro methods to address oxidative stress and nanoparticle-specific mechanisms [19,20,21]. Meanwhile, the transition from 2D monolayers to three-dimensional (3D) culture systems improved physiological relevance and predictive accuracy [22].

Recent technological progress has transformed the scope and ambition of cytotoxicity testing. Organoids derived from pluripotent stem cells now reproduce the complex architecture and functionality of native tissues, improving the prediction of hepatotoxicity, nephrotoxicity, and ocular or dermal injury [23,24,25]. Microfluidic organ-on-chip systems allow dynamic investigation of absorption, distribution, metabolism, and excretion (ADME), and provide mechanistic insight into tissue–tissue interactions and systemic responses [26,27,28]. Alongside these in vitro innovations, computational tools—such as quantitative structure–activity relationship (QSAR) modelling, machine learning, and physiologically based pharmacokinetic (PBPK) or quantitative in vitro–in vivo extrapolation (QIVIVE) models—now enable quantitative translation of laboratory data into realistic human exposure scenarios [29,30,31,32].

Together, these advances form the foundation of New Approach Methodologies (NAMs) and Integrated Approaches to Testing and Assessment (IATA), which are now being incorporated into OECD, FDA, and EMA regulatory frameworks [6,33,34]. Validated 3D skin and corneal models have already replaced traditional animal-based assays such as the Draize test [33,35,36].

Overall, the evolution of cytotoxicity testing reflects a clear shift from simple viability measurements to integrated, mechanistic, and human-relevant systems. These new platforms bridge experimental biology, computational modelling, and regulatory science, advancing both the predictive power and ethical sustainability of toxicological assessment. The present review provides a comprehensive overview of this transition—from classical in vitro assays to high-throughput, stem cell-based, in silico, and organ-on-chip technologies—and discusses their growing significance for biomedical research and regulatory decision making. Unlike earlier reviews that focused primarily on classical cytotoxicity assays [12,37,38] or selectively on emerging 3D and microphysiological systems [39,40,41], the present article integrates these methodological domains with advanced organoid platforms, stem cell-based models, and contemporary in silico NAM/IATA frameworks, providing a unified and mechanistically oriented perspective on modern cytotoxicity testing.

**Figure 1:**
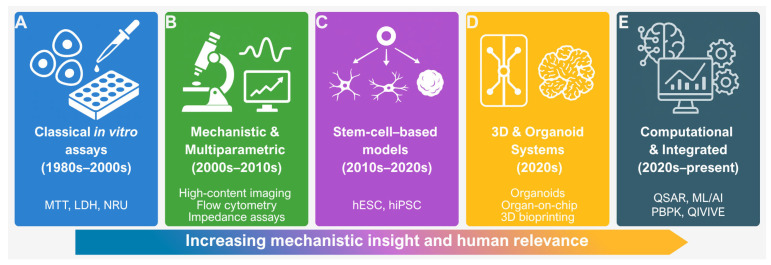
Overview of the evolution of cytotoxicity testing, from classical in vitro assays to mechanistic, stem cell–based, 3D/organoid, and computational approaches. Panels (**A**–**E**) illustrate the progressive increase in mechanistic insight and human relevance across these methodological categories.

An overview of this methodological evolution is illustrated in Figure 1.

## 2. Classical Cytotoxicity Assays: Foundations of In Vitro Toxicology

**Figure 2:**
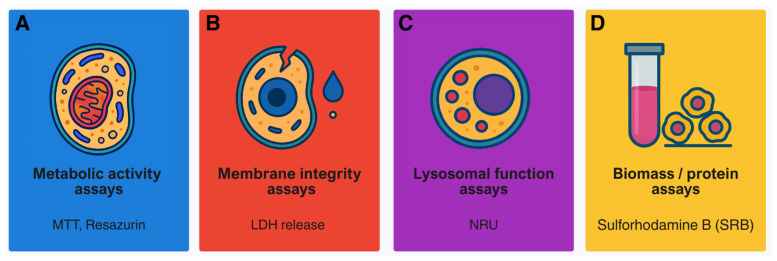
Overview of major functional categories of classical cytotoxicity assays used as foundational endpoints in in vitro toxicology. (**A**) Metabolic activity assays (e.g., MTT, resazurin) quantify mitochondrial and cytosolic reduction capacity as an indicator of cell viability. (**B**) Membrane integrity assays (e.g., LDH release) detect loss of plasma membrane integrity associated with necrotic or late-stage cytotoxicity. (**C**) Lysosomal function assays (e.g., neutral red uptake, NRU) measure lysosomal accumulation of neutral dyes to assess early lysosomal stress. (**D**) Biomass/protein assays (e.g., sulforhodamine B, SRB) quantify total cellular protein content as a metabolism-independent indicator of cell number.

Classical cytotoxicity assays remain a fundamental component of in vitro toxicology and continue to provide accessible, reproducible, and straightforward means to evaluate cell viability and detect toxic responses. Their place in the broader historical development of the field is illustrated in Figure 1A, where they represent the earliest generation of in vitro testing strategies. Despite well-recognised limitations in mechanistic depth and physiological relevance, these assays persist as essential reference points in both research and regulatory contexts. As shown in Figure 2, classical methods can be classified according to their primary biological endpoint. Among the most widely applied assays are tetrazolium reduction (MTT), LDH release, NRU, resazurin reduction, and total protein or biomass quantification. Together, they form the practical foundation of routine cytotoxicity assessment, offering a balance between simplicity and reliability.

To ensure data quality and comparability, careful attention to assay design, appropriate controls, and transparent data reporting is critical. Key recommendations for good experimental practice—including validation steps, interference checks, and result normalisation—are summarised in Box 1.

Box 1 **Best practices for classical cytotoxicity assays.**

**Assay design and execution**

- Verify signal linearity with cell density (5 × 10^3^–2 × 10^4^ cells/well in 96-well plates);
- Optimise dye incubation times (e.g., 2–4 h MTT; 3 h NRU) and report conditions;
- Control for LDH background in serum; use serum-free or heat-inactivated controls.

**Controls and interference checks**

- Screen test compounds for intrinsic fluorescence or colour; include “no-cell” blanks;
- Assess dye adsorption by nanomaterials and confirm with independent endpoints;
- Use appropriate positive and negative controls (e.g., Triton X-100, staurosporine) to verify responsiveness.

**Data processing and normalisation**

- Subtract background from blank wells;
- Normalise viability to untreated controls (100%) and maximal lysis (0%);
- Report raw data, at least three biological replicates with technical triplicates, and variability metrics.

**Reporting transparency**

- Specify seeding density, passage number, medium composition, incubation time, dye concentration, and detection settings;
- Describe curve fitting and statistical methods clearly;
- Note any deviations from OECD or ISO guidelines.

Tetrazolium-based assays such as MTT estimate cellular metabolic activity by monitoring the enzymatic reduction in tetrazolium salts into insoluble formazan crystals through mitochondrial and cytosolic dehydrogenases (Figure 1A). Long regarded as the “gold standard” for assessing cytotoxicity, the MTT assay is now known to be susceptible to several artefacts, including non-specific reduction by test compounds or medium components, non-linear responses to cell number, and difficulties in formazan solubilisation [12,42]. As a result, data derived from MTT assays should be interpreted with caution and ideally confirmed using independent endpoints.

LDH release assays offer a more direct measure of plasma membrane integrity by quantifying extracellular enzyme activity through coupled colorimetric reactions (Figure 1B). Their simplicity makes them attractive for routine use, yet accuracy can be affected by serum background, spontaneous leakage from stressed cells, or chemical interference. Combining LDH measurements with complementary readouts—such as proliferation or cell cycle analyses—enhances the mechanistic interpretation of cytotoxic effects [10,43,44].

The NRU assay evaluates lysosomal function by measuring the accumulation of neutral red dye in viable cells (Figure 1C). Compared with MTT, NRU is often more sensitive to early lysosomal stress, but results can be influenced by pH, incubation time, or lysosomal stability [11,45].

Resazurin (alamarBlue) reduction provides a non-destructive metabolic endpoint that allows repeated, same-well measurements and long-term monitoring (Figure 1A). It is typically more sensitive and less variable than MTT or LDH, although very high metabolic activity can cause premature signal saturation [46,47].

Protein- and biomass-based assays such as sulforhodamine B (SRB) or Bradford staining quantify total cellular mass independently of metabolism (Figure 1D). When used alongside metabolic assays, they help differentiate cytostatic from cytotoxic effects and reduce the risk of misinterpretation [48,49].

Comparative analyses consistently show that no single assay provides universally reliable results. NRU and resazurin methods often detect early toxicity more effectively, whereas MTT and LDH may underestimate subtle effects or yield higher variability [46]. Experimental artefacts—such as medium composition influencing dye uptake or nanoparticle adsorption of chromogenic reagents—can further confound outcomes [50,51,52]. Consequently, multiparametric strategies combining at least two independent endpoints are now considered best practice [48,53]. Common sources of error and assay-specific pitfalls are summarised in Box 2.

Beyond their technical advantages, classical cytotoxicity assays continue to play a key role in regulatory and screening contexts. In large-scale initiatives such as ToxCast, non-specific cytotoxicity signals often dominate and can obscure mechanistic responses [15]. Viability endpoints, therefore, remain indispensable for normalising data and guiding interpretation [44]. Even in advanced 3D organoid and spheroid models, commercially available cytotoxicity kits frequently show inter-donor or inter-platform variability, highlighting both the ongoing relevance of these classical assays and the need for multiparametric calibration [54]. Within tiered testing frameworks, they typically represent the first step of evaluation, preceding mechanistic, high-content, or omics-based analyses [55].

Box 2 **Common pitfalls in classical cytotoxicity assays.**

- MTT: non-specific reduction by compounds or medium; insoluble formazan crystals; metabolic stimulation mistaken for viability [42];
- NRU: dependence on pH or lysosomal health; false cytotoxicity when lysosomes are targeted [45];
- LDH release: serum background, spontaneous leakage, chemical interference [43,44].
- Resazurin: over-reduction in highly active cells; fluorescence quenching by test compounds [47];
- Protein/biomass assays: variability in fixation or staining; insensitivity to metabolic suppression without cell loss [49];
- General: use of a single endpoint; nanoparticle interference; incomplete reporting [48,52,54].

## 3. Transition from Viability Endpoints to Mechanistic Approaches

As summarised in Figure 1B, mechanistic and multiparametric assays expanded toxicological readouts beyond simple viability endpoints. The limitations of single-endpoint viability assays have become increasingly apparent in contemporary toxicology. For many years, methods such as MTT, LDH, and NRU provided reliable first-tier screening tools, yet their reductionist perspective captured only a narrow view of cellular injury. As research and regulation have moved towards more human-relevant, mechanistic, and predictive approaches, cytotoxicity testing has undergone a marked transformation. The discipline now embraces high-throughput, multiparametric, and physiologically relevant models that provide richer and more quantitative insight into cellular responses. This evolution represents more than a technical improvement—it signifies a conceptual shift in how toxicity is understood, measured, and integrated into risk assessment.

### 3.1. From Viablity to High-Throughput Screening

A decisive step forward came with the introduction of quantitative high-throughput screening (qHTS) technologies, developed through large-scale programmes such as Tox21 [16] and ToxCast [56]. These initiatives screened thousands of industrial chemicals, pharmaceuticals, and environmental contaminants in miniaturised 1536-well formats, producing extensive datasets that captured activity across nuclear receptor, stress response, and enzyme inhibition pathways [57,58]. One clear lesson emerged from these studies: broad cytotoxicity effects often dominated assay outcomes, obscuring genuine pathway-specific responses and complicating interpretation. For instance, in oestrogen receptor reporter assays, apparent activity was frequently driven by non-monotonic dose–response curves or secondary stress effects rather than by true receptor engagement [59].

To prevent such misinterpretation, viability assays were incorporated into Tox21 [16] and ToxCast [56], not as primary endpoints but as parallel counterscreens. In practice, this meant that compounds showing apparent mechanistic activity were re-examined for their impact on cell viability: if the signal loss coincided with reduced viability, the response was classified as artefactual [57,60]. Mechanistic studies using dendrimers provided a concrete example—demonstrating that elevated cytotoxicity was accompanied by apoptotic signalling and TRAIL-mediated cell death in chronic lymphocytic leukaemia models [61].

Standardised data analysis pipelines also improved reproducibility. The ToxCast Pipeline for Curve Fitting (tcpl) and curated curve classification schemes [56,62] enabled consistent handling of replicates and background correction. Collectively, these developments transformed high-throughput screening from a largely descriptive exercise into a mechanistically anchored, data-driven paradigm-laying the foundation for truly multiparametric approaches.

### 3.2. Multiparametric and High-Content Imaging Approaches

A second wave of innovation in cytotoxicity testing was driven by the rise of high-content imaging (HCI) and phenotypic profiling technologies, which were designed to move beyond binary viability readouts. HCI integrates automated microscopy with quantitative image analysis, capturing a wide range of cellular features—nuclear and mitochondrial morphology, lysosomal integrity, cytoskeletal organisation, and more—across thousands of individual cells [63,64]. These multiparametric datasets reveal early, adaptive, or sub-lethal effects that conventional endpoint assays often miss. Among the most influential frameworks, the Cell Painting assay combines multiplexed fluorescent labelling of key organelles with computational feature extraction to generate morphological fingerprints that cluster structurally diverse compounds by their mechanisms of action [65,66].

When paired with transcriptomic or metabolomic profiling, these morphological signatures enable predictive modelling of toxicity pathways. Early comparative studies demonstrated that imaging-based cytotoxicity assays combined with proliferation markers were more sensitive than classical metabolic assays such as MTT or LDH [63]. Specialised variants have since emerged, including the BlueScreen HC genotoxicity assay, which incorporates DNA damage reporters [67], and microglia-focused imaging platforms that quantify phagocytosis and cell health [63]. Crucially, these systems can detect adaptive stress responses well below overt cytotoxic thresholds, offering a window into the early stages of cell injury.

Phenotypic profiling has therefore transformed cytotoxicity assessment from a descriptive measure of cell death into a mechanistic discipline at the interface of cell biology, cheminformatics, and predictive toxicology [68].

Beyond imaging, impedance-based systems such as the xCELLigence Real-Time Cell Analysis (RTCA) platform provide continuous, label-free monitoring of proliferation, adhesion, and cell death. By recording time-resolved fluctuations in cell index, RTCA distinguishes transient stress from irreversible damage, capturing subtle morphological dynamics that static assays like MTT cannot resolve [69,70,71].

Flow cytometry represents another cornerstone of multiparametric cytotoxicity analysis, allowing simultaneous detection of apoptosis, mitochondrial depolarisation, and oxidative stress at the single-cell level [72]. Fluorescence-based measurements of Annexin V/Propidium Iodide (PI) staining, reactive oxygen species (ROS), and caspase activation provide detailed mechanistic information [73,74]. Because fluorescence detection is largely unaffected by nanoparticle interference, flow cytometry remains particularly valuable where optical artefacts compromise colorimetric assays [75,76].

Confocal microscopy complements these approaches by visualising intracellular compound distribution, including fluorescent nanocarriers, and identifying organelle-specific accumulation linked to oxidative stress or membrane disruption [75,76]. Integrated live cell confocal imaging adds a spatial dimension, confirming the localisation of toxicity pathways and supporting the mechanistic classification of compound-induced injury.

Together, these multiparametric and real-time analytical methods have redefined the purpose of cytotoxicity testing. Rather than serving merely to identify toxic hits, they now function as mechanistic tools that illuminate the pathways of cellular perturbation, bridging discovery toxicology with systems biology.

### 3.3. Refining Genotoxicity Assays to Reduce False Outcomes

Despite decades of routine use, in vitro genotoxicity assays have long struggled with issues of reproducibility and specificity. High false-positive rates—particularly in p53-deficient rodent cell lines—have frequently resulted in costly and unnecessary follow-up investigations [13]. Over the past decade, however, a series of methodological refinements has greatly improved both the human relevance and reliability of these assays.

One of the most effective improvements has been the use of p53-competent human cell lines, which significantly reduce spurious DNA damage responses [77]. In addition, the introduction of refined viability parameters such as Relative Population Doubling (RPD) and Relative Increase in Cell Counts (RICC) has enhanced the ability to distinguish between general cytotoxicity and genuine genotoxicity [78]. Comparative studies consistently show that human cell lines respond more predictably to known genotoxicants than rodent systems, further supporting their use in human-relevant testing strategies [79].

In parallel, orthogonal high-throughput assays such as the CometChip—a microarray adaptation of the classical comet assay—have enabled the simultaneous quantification of DNA strand breaks across hundreds of samples [80]. By combining scalability with mechanistic endpoints, platforms like CometChip bridge the gap between traditional low-throughput tests and regulatory applications, reducing both false-positive and false-negative outcomes.

Together, these advances illustrate how improved methodological precision and mechanistic understanding can resolve many of the historical weaknesses of in vitro genotoxicity testing, strengthening its role in modern toxicological assessment

### 3.4. Bridging to Three-Dimensional Cultures and Organoids

A major turning point in cytotoxicity testing has been the emergence of 3D cultures and organoids, which more accurately reproduce tissue architecture, cell–cell communication, and physiological gradients absent from traditional 2D monolayers. By better reflecting the structural and functional complexity of native tissues, 3D systems offer markedly improved translational relevance and predictive power.

Co-culture spheroid models that integrate tumour and immune cells now enable real-time monitoring of immune-mediated cytotoxicity using luminescence-based killing assays and multicolour flow cytometry [81,82]. Comparable approaches have been applied to evaluate CAR-T cell–induced cytotoxicity in high-throughput settings, providing reproducible platforms for immunotoxicity research [83]. Microfluidic systems such as CACI-IMPACT further enhance these models by allowing continuous perfusion and kinetic imaging of cytotoxic responses under dynamic flow conditions [84].

Beyond oncology, the use of 3D neuronal spheroid cultures, such as LUHMES-derived models, facilitates mechanistic neurotoxicity screening [85], while liver organoids and liver-on-chip platforms surpass conventional 2D hepatocyte assays in predicting drug-induced liver injury [86,87]. Comparative proteomic and transcriptomic analyses consistently demonstrate distinct mechanistic signatures between 2D and 3D models, highlighting the superior physiological fidelity of the latter [88,89,90].

Together, these innovations bridge classical in vitro testing with in vivo relevance and align closely with the principles of NAMs and IATA [91]. Within the IATA framework, 3D cultures and organoids are increasingly integrated with in silico models, microphysiological systems, and omics datasets to provide human-relevant, non-animal evidence for toxicity evaluation [92]. Defined Approaches (DAs) combine outcomes from 3D assays with QSAR or read-across models, while PBPK and QIVIVE modelling translate in vitro activity into human-equivalent doses [93,94].

Practical recommendations for experimental design and quality control during this transition are summarised in Box 3.

Box 3 **Practical guidance for modern in vitro cytotoxicity studies.**

- Use viability as a counterscreen, not as an endpoint: include viability to flag artefacts rather than as the main signal; apply standardised pipelines such as tcpl and curve classification workflows [56,62];
- Adopt multiparametric imaging: employ HCI and Cell Painting to capture sub-lethal mechanisms and assist hit triage [64,66,95];
- Improve genotoxicity reliability: use p53-competent human cells, RPD/RICC thresholds, and orthogonal assays such as CometChip [77,78,79,80];
- Increase physiological relevance: integrate 3D spheroids and organoids (including immune co-cultures and liver models) with functional biomarkers and, where possible, omics data [81,82,83,84,85,86,87,88,89,90,96].

## 4. Stem Cell-Based Models in Cytotoxicity Testing

Stem cell-based models occupy the next stage of methodological development (Figure 1C), providing human-relevant systems for developmental and organ-specific toxicity. Pluripotent stem cell-based models have become indispensable to modern cytotoxicity testing, providing human-relevant systems that overcome many of the limitations associated with immortalised tumour cell lines. Both hESCs and hiPSCs possess the remarkable capacity to differentiate into virtually any cell type, offering a unique platform to study xenobiotic effects across different developmental stages and organ systems [97,98,99]. By enabling controlled differentiation into functionally mature cells, these models allow researchers to investigate cellular mechanisms underlying toxicity within a physiologically meaningful human context.

Together, hESC- and hiPSC-derived systems have transformed toxicology from largely descriptive testing into a mechanistic science—one capable of linking cellular perturbations to adverse outcomes with unprecedented relevance for human health.

### 4.1. Human Embryonic Stem Cells (hESCs)

hESCs are derived from the inner cell mass of pre-implantation blastocysts, typically obtained from surplus embryos generated during in vitro fertilisation and donated with informed consent [100]. Their inherent pluripotency and virtually unlimited capacity for self-renewal make them a powerful platform for generating physiologically relevant human cell types in vitro. Over the past decade, refined differentiation protocols have enabled the establishment of diverse hESC-based models suited for organ-specific cytotoxicity testing [97,99].

Among these, hESC-derived cardiomyocytes have become benchmark systems for detecting drug-induced cardiotoxicity and contractility disturbances [101], while hepatocyte-like cells are increasingly employed to evaluate metabolism-dependent hepatotoxicity and drug-induced liver injury [102,103]. Neural and neuronal progenitor lineages derived from hESCs are widely used in developmental neurotoxicity studies [104,105], and epithelial derivatives from intestinal or pulmonary differentiation pathways now serve as human-relevant models for assessing mucosal and respiratory toxicity [106,107].

At a mechanistic level, transcriptomic and epigenetic profiling of differentiating hESCs exposed to environmental or pharmaceutical toxicants has revealed characteristic developmental hazard signatures [97]. These reproducible molecular fingerprints—defined by changes in gene expression and chromatin regulation—emerge when early developmental processes are perturbed. Model teratogens such as methylmercury, valproic acid, and all-trans retinoic acid elicit distinct transcriptional responses associated with neurogenesis, morphogenesis, and retinoid signalling. Such molecular fingerprints provide mechanistic evidence of early embryotoxicity and enable quantitative discrimination between toxic and non-toxic compounds. This level of mechanistic resolution demonstrates how hESC-based assays bridge molecular perturbations with adverse developmental outcomes, advancing predictive toxicology beyond descriptive endpoints.

Despite their scientific advantages, the use of hESC-based systems remains ethically and legally constrained in many regions. Access to approved lines is limited, and differentiated derivatives can exhibit partial immunogenicity [108]. Moreover, the persistence of residual undifferentiated cells presents a teratoma formation risk, requiring rigorous purification and quality control measures [109,110].

Overall, hESCs have laid the conceptual and methodological foundation for human developmental toxicity testing. Yet, their ethical and technical constraints have spurred the development of reprogrammed alternatives such as hiPSCs, which now represent a versatile and ethically sustainable next step in predictive toxicology.

### 4.2. Induced Pluripotent Stem Cells (hiPSCs)

HiPSCs are generated by reprogramming adult somatic cells—most commonly fibroblasts, peripheral blood mononuclear cells, or urinary epithelial cells—through enforced expression of the Yamanaka factors OCT4, SOX2, KLF4, and c-MYC [18,98,111,112]. This discovery revolutionised stem cell biology and provided an ethically acceptable, patient-specific alternative to embryonic material.

Since their introduction, hiPSC-based models have become integral to contemporary toxicology. Among their most established applications are hiPSC-derived cardiomyocytes (hiPSC-CMs), which faithfully reproduce human electrophysiological and contractile properties and have proven highly effective for predicting chemotherapy- and drug-induced cardiotoxicity [101,113,114]. Hepatocyte-like cells and liver organoids derived from hiPSCs recapitulate key metabolic and cholestatic functions, enabling high content and mechanistic evaluation of drug-induced liver injury [115,116]. Similarly, neuron-rich cultures and cerebral organoids have become essential tools for developmental neurotoxicity studies, capturing molecular and structural alterations caused by teratogens such as thalidomide [117] and environmental pollutants like perfluorooctanoic acid [118].

Beyond these core applications, hiPSCs enable the development of renal and ocular toxicity models—including nephron-like constructs [119] and retina-on-a-chip systems [120]—as well as complex multi-lineage organoids for studying cross-tissue interactions, such as combined cardiac–hepatic or tumour–microenvironment responses [121]. Because they retain the genetic background of the donor, hiPSC-based systems also support the emerging field of precision toxicology, allowing inter-individual susceptibility to xenobiotics to be explored directly in vitro.

Despite these advantages, challenges remain. Inter-line variability, incomplete cellular maturation, and batch-dependent differences in differentiation efficiency continue to limit reproducibility and regulatory acceptance [122]. Addressing these issues will be essential for the routine implementation of hiPSC-based assays in safety assessment pipelines.

In summary, hiPSCs combine ethical acceptability with patient specificity, providing a versatile bridge between mechanistic toxicology and personalised medicine.

### 4.3. Applications in Developmental and Organ-Specific Toxicity

The introduction of pluripotent stem cell–based assays has reshaped developmental toxicity testing, driving a shift from animal teratogenicity models to human-relevant, mechanistically anchored systems. Integrated platforms such as PluriLum and ReproTracker combine controlled PSC differentiation with transcriptomic, proteomic, and imaging readouts to quantitatively map teratogenic signatures. When integrated with PBPK modelling, these assays enable QIVIVE, substantially improving the prediction of human developmental hazards [123,124].

Organ-specific applications have expanded across multiple tissue types, bringing a level of physiological depth previously unattainable in in vitro cytotoxicity testing. In the cardiovascular field, hiPSC-CMs and 3D cardiac organoids have become cornerstone systems for assessing electrophysiological and structural cardiotoxicity. These models reproduce key human myocardial properties—including action potential dynamics, calcium handling, and contractility—allowing quantitative evaluation of pro-arrhythmic and cardio-depressive effects often missed in animal studies [113,125]. The integration of microelectrode array and optical mapping technologies further enhances predictive power by linking electrophysiological readouts to mitochondrial and molecular stress responses.

In hepatotoxicity research, hPSC-derived hepatocytes and liver organoids now serve as advanced platforms for evaluating xenobiotic metabolism and drug-induced liver injury. Their metabolic competence—including cytochrome P450 activity—together with 3D tissue organisation enables the detection of both acute hepatocellular damage and delayed cholestatic responses [115,116]. The presence of structured bile canaliculi and polarised hepatocytes allows realistic modelling of bile secretion and transporter-mediated toxicity, bridging the long-standing gap between in vitro and in vivo hepatic physiology.

Within the nervous system, PSC-derived brain organoids and neuron-enriched spheroids have become pivotal tools for studying developmental neurotoxicity and neurodegenerative mechanisms. These self-organising 3D cultures reproduce regional brain patterning, cortical layering, and synaptic maturation, making them uniquely suited to analyse neurodevelopmental disruption caused by teratogens or environmental chemicals [126,127]. Exposure studies in these models reveal alterations in neuronal differentiation, synaptogenesis, and glial–neuronal communication-mechanistic endpoints that remain inaccessible to traditional 2D assays.

Renal models derived from hiPSCs have also progressed rapidly. Nephron-like organoids and bioprinted kidney constructs reproduce key aspects of renal filtration and tubular transport, allowing detailed analysis of nephrotoxicity and tubular injury [119,127]. These constructs express segment-specific markers and support compound accumulation and transport studies, offering a more physiologically relevant alternative to immortalised renal cell lines.

Taken together, pluripotent stem cell–derived organoids and organ-on-chip systems mark a decisive step towards integrated, multi-tissue toxicology. By connecting metabolic, electrophysiological, and developmental endpoints, these platforms enhance the predictive accuracy of in vitro assays and bring toxicology closer than ever to truly human-relevant models.

### 4.4. Ethical and Technical Considerations

Research involving human stem cells continues to raise both ethical and technical challenges. Work with hESCs, derived from early-stage embryos, remains one of the most tightly regulated areas of biomedical science. In countries where hESC research is permitted, stringent oversight mechanisms govern every stage of the process, ensuring donor consent, traceability, and compliance with recognised bioethical standards. Foundational documents such as the Declaration of Helsinki [128] and the EU Directive 2004/23/EC on Human Tissues and Cells [129] outline donor protection principles across the European Union, while the National Institutes of Health (NIH) Stem Cell Registry [130] defines which hESC lines qualify for federally funded research in the United States. On a global level, the International Society for Stem Cell Research (ISSCR) Guidelines for Stem Cell Research and Clinical Translation (2021) provide a comprehensive ethical framework that addresses informed consent, genomic data protection, and the prohibition of reproductive cloning [131,132].

Although hiPSCs avoid embryo-related ethical concerns, they introduce a different set of issues. Donor privacy, consent for genomic data use, and the potential misuse of reprogramming technologies for reproductive purposes have all become areas of ethical scrutiny [131,133,134,135,136,137]. The expanding number of patient-specific hiPSC lines underscores the need for robust governance in biobanking, data sharing, and genomic security [133].

From a technical perspective, pluripotent stem cell–based models still face several challenges, particularly regarding reproducibility, inter-line variability, and incomplete maturation of differentiated derivatives. These factors can affect predictive accuracy and hinder regulatory validation. Substantial progress has been made through the development of automated culture systems, lineage-specific fluorescent reporters, and high-content phenotyping pipelines that help reduce variability and improve throughput [138,139]. Nevertheless, rigorous quality control remains essential: residual undifferentiated cells must be excluded, as both hESC- and hiPSC-derived products carry an inherent risk of teratoma formation [110,140].

As ethical frameworks become increasingly harmonised and technical refinements continue to improve reliability, PSC-based models are gaining broader recognition as both scientifically robust and ethically sound platforms for regulatory toxicology.

For clarity, the principal ethical frameworks and procedural requirements for stem cell research are summarised in Box 4.

Box 4 **Ethical frameworks for stem cell toxicity models.**

- **Declaration of Helsinki** (2013)—Universal ethical principles for research involving human-derived material; mandates informed consent and independent ethical review [128];
- **EU Directive 2004/23/EC**—Standards for donor consent, traceability, and supervision across EU member states [129];
- **NIH Stem Cell Registry (United States)**—Specifies approved hESC lines for federally funded research in the US [130];
- **ISSCR Guidelines for Stem Cell Research and Clinical Translation (2021)**—Global reference for hESC/hiPSC research; emphasises informed consent, data protection, and prohibition of reproductive cloning [131];
- **National and Institutional Oversight Committees**—Ensure compliance with local ethical regulations [131].

**Practical requirements:**

1. Documented donor consent (in vitro fertilisation (IVF) or somatic cell source);
2. Registration of cell lines in recognised repositories;

Institutional ethics board approval and adherence to ISSCR guidance.

### 4.5. Adult Stem Cell Models

In addition to pluripotent stem cell systems, adult stem cells provide valuable complementary tools for cytotoxicity testing, particularly when tissue-specific or immunological endpoints are of interest. Among these, haematopoietic stem cells (HSCs) and mesenchymal stromal cells (MSCs) derived from bone marrow are the most widely applied [141].

Human MSCs have also been employed to investigate the cytotoxicity and biocompatibility of photobiomodulation procedures. Measurements of viability, calcium signalling, and oxidative balance revealed a pronounced sensitivity of MSCs to irradiation parameters [142]. Interestingly, subsequent studies showed that sub-lethal photobiomodulation doses could enhance proliferation and maintain stemness, suggesting that stem cell–based toxicity models are capable of detecting adaptive, even beneficial, stress responses [142].

HSCs, on the other hand, have long served as highly sensitive indicators of bone marrow toxicity. Their intrinsic ability to form distinct haematopoietic colonies in vitro forms the basis of the colony-forming unit (CFU) assay, which quantifies progenitor survival and differentiation following exposure to xenobiotics or chemotherapeutic agents [143,144]. These assays provide direct mechanistic insight into myelotoxic and immunosuppressive effects that often mirror the clinical manifestations of haematopoietic injury [145].

MSCs isolated from bone marrow, adipose tissue, or umbilical cord have also gained prominence in evaluating the cytotoxicity and biocompatibility of biomaterials, nanomaterials, and regenerative scaffolds [146,147]. Their multipotent capacity to differentiate into osteogenic, chondrogenic, and adipogenic lineages enables mechanistic assessment of toxicant-induced alterations in bone, cartilage, and connective tissue physiology [148]. Notably, MSC-based assays have become essential for evaluating the safety of medical implants and nanoparticles, effectively linking toxicology with biomaterials science and regenerative medicine [149].

Compared with pluripotent stem cells, adult stem cell–based models are more accessible and ethically straightforward, making them particularly well suited for targeted, tissue-specific investigations. However, their limited differentiation potential and donor-dependent variability restrict their broader application in mechanistic or high-throughput toxicology [150]. They therefore occupy a complementary niche—providing valuable insight into immunotoxicity, myelotoxicity, and biomaterial compatibility—while hESC- and hiPSC-based systems remain the principal human-relevant platforms for comprehensive cytotoxicity evaluation.

The comparative features of pluripotent and adult stem cell models, including their sources, differentiation potential, applications, and ethical considerations, are summarised in Table 1.

In concert, pluripotent and adult stem cell platforms unite ethical and scientific strengths, advancing human-relevant, mechanistically informed, and personalised toxicity testing for next-generation safety assessment.

## 5. Nanotoxicology and Specialised In Vitro Models

Building on the mechanistic insights gained from stem cell–based systems, nanotoxicology applies similar principles to examine how nanoscale materials interact with biological environments. Modern nanotoxicology increasingly prioritises mechanistic and physiologically relevant assessment over traditional, purely descriptive viability testing. Across diverse classes of engineered nanomaterials—including metallic, oxide, and polymeric types—oxidative stress has emerged as a central initiating mechanism driving downstream inflammatory and cytotoxic responses [151,152,153,154].

Recent refinements of classical cytotoxicity assays, coupled with the integration of 3D cultures, microfluidic platforms, and stem cell–based systems, have established a more predictive and human-relevant framework for nanotoxicology. These advances link the physicochemical properties of nanoparticles to molecular and cellular perturbations, bridging nanoscale structure with biological function and enabling more accurate assessment of human health risks [155,156,157,158].

### 5.1. Cytotoxicity of Nanomaterials: Mechanistic Basis of Oxidative Stress

Engineered nanomaterials interact with biological systems through distinctive physicochemical properties that can disrupt redox balance and trigger oxidative stress. Among the most consistent mechanisms of nanoparticle-induced cytotoxicity is the excessive generation of ROS, which impairs mitochondrial function, damages DNA, and activates pro-inflammatory signalling pathways. Metal oxides such as ZnO, TiO_2_, Fe_2_O_3_, and CeO_2_ readily catalyse ROS formation through surface redox reactions and electron transfer, leading to lipid peroxidation, mitochondrial dysfunction, and apoptotic cell death [151,152,153,159]. The magnitude of oxidative injury depends strongly on particle size, surface charge, aggregation behaviour, and the composition of the surrounding protein corona [160,161,162].

Similar redox-driven mechanisms have been observed with polymeric nanostructures such as dendrimers, where a high surface charge density can induce mitochondrial depolarisation, caspase activation, and oxidative DNA damage [154,163]. Surface functionalisation with neutral or carbohydrate groups markedly reduces ROS generation and helps restore cellular redox homeostasis [160,164]. Collectively, these findings support a unifying model in which nanoparticle toxicity arises primarily from physicochemical interactions that overwhelm endogenous antioxidant defences, rather than from the intrinsic chemical composition of the material.

Beyond direct oxidative damage, nanomaterial exposure can also provoke a spectrum of secondary stress responses—including inflammasome activation, mitophagy, and stress granule formation—largely driven by ROS-dependent signalling [165,166]. Together, these interconnected processes establish oxidative stress as a central initiating event that links nanoparticle physicochemistry to downstream pathways of apoptosis, inflammation, and adaptive stress responses.

### 5.2. Adaptation of Classical Cytotoxicity Assays to Nanomaterials

Traditional viability assays—such as MTT, LDH release, NRU, and resazurin reduction—were originally developed for testing soluble compounds and often yield unreliable results when applied to nanomaterials. Nanoparticles can adsorb assay dyes, scatter incident light, or catalyse redox reactions, resulting in false-positive or false-negative outcomes [75,167]. Comparative studies have consistently shown that no single assay endpoint can reliably capture nanoparticle-induced toxicity, and that optical interference remains a major source of experimental variability [76,167].

To address these limitations, current best practices emphasise careful control of experimental design, including the use of nanoparticle-only controls, orthogonal readouts, and thorough characterisation of dispersion state, serum content, and incubation time [168]. Studies using cationic polymer nanocarriers have demonstrated that many apparent cytotoxic effects stem from interactions with assay reagents rather than genuine cellular injury. In such cases, alternative assays—focusing on membrane integrity or haemolytic activity—can offer more reliable indicators of nanoparticle-induced damage [169,170].

Overall, these refinements highlight that classical viability assays alone are insufficient for accurate nanotoxicity assessment. Incorporating mechanistic endpoints—such as ROS quantification, mitochondrial membrane potential, or caspase activation—provides a more robust and interpretable evaluation of nanoparticle-induced cellular effects.

### 5.3. Specialised In Vitro Models and Specific Endpoints

Advances in in vitro methodology have transformed nanotoxicology from a largely descriptive discipline into one grounded in mechanistic understanding and physiological relevance. Three-dimensional cultures, organoids, and microfluidic “organ-on-chip” platforms now replicate native tissue architecture and biochemical gradients, substantially improving the predictive power of in vitro testing. For example, liver spheroids and organoid models preserve metabolic competence and reveal delayed hepatotoxic effects that remain undetectable in conventional monolayer cultures [96,157]. The pioneering lung-on-a-chip system introduced dynamic cyclic strain and epithelial–endothelial co-cultures to mimic nanoparticle deposition at the air–liquid interface [158], while subsequent intestine- and skin-on-chip designs have enabled real-time monitoring of barrier integrity and inflammatory mediator release [156,171].

Stem cell–derived and bioprinted organoids have further expanded nanosafety assessment into developmental and regenerative contexts, supporting long-term studies of sublethal toxicity and adaptive stress responses [155,172]. Mechanistic endpoints—such as ROS production, mitochondrial membrane potential, NF-κB activation, and cytokine release—are now routinely measured alongside classical viability assays, yielding a multidimensional picture of nanoparticle–cell interactions [164,165,173].

Together, these technological advances integrate detailed physicochemical characterisation with systems-level biology, establishing a comprehensive, mechanistically anchored, and human-relevant framework for nanosafety evaluation.

Key mechanistic principles and methodological standards of modern nanotoxicology are summarised in Box 5.

Box 5 **Practical and mechanistic insights into nanotoxicology.**

- **Mechanistic basis:** Oxidative stress and the overproduction of ROS are the primary initiators of nanoparticle-induced toxicity. These processes trigger apoptosis, inflammasome activation, and NF-κB signalling, linking physicochemical properties with cellular injury [151,153,154,166].
- **Assay adaptation:** Nanoparticles interfere with colorimetric and fluorometric assays by adsorbing dyes or catalysing redox reactions. Reliable assessment therefore requires nanoparticle-only controls and confirmation using orthogonal endpoints such as ATP quantification or impedance-based measurements [75,76,167,170].
- **Physiological relevance:** Advanced in vitro models—3D spheroids, organoids, and organ-on-chip platforms—reproduce tissue-level gradients and dynamic perfusion, thereby improving correlation with in vivo outcomes [156,157,158,172].
- **Functional readouts:** Mechanistic biomarkers such as ROS levels, mitochondrial potential, and cytokine release reveal sublethal and adaptive stress responses that conventional viability assays may overlook [164,165,173].
- **Standardisation:** Harmonised experimental conditions, transparent reporting, and mechanistic mapping enhance reproducibility and regulatory acceptance of nanosafety data [168,174].

## 6. Advanced 3D Models: Organoids, Organ-on-Chip, and Bioprinting

Recent advances in bioengineering and microphysiological systems (MPSs) have profoundly reshaped in vitro toxicology, providing human-relevant models that reproduce organ-level functions and systemic pharmacokinetics. Three-dimensional organoid cultures, microfluidic organ-on-chip devices, and bioprinted tissues now capture essential features of native tissue organisation—such as perfusion, polarisation, and intercellular communication—that were long unattainable in conventional monolayers. By generating quantitative, mechanism-based data that link cellular perturbations to tissue- and organism-level outcomes, these systems effectively bridge the gap between molecular assays and clinical toxicology. Figure 1D highlights the emergence of 3D and MPSs that capture tissue architecture and dynamic perfusion.

The incorporation of dynamic flow, multi-organ coupling, and computational integration within these platforms represents a decisive step towards predictive toxicology that aligns with emerging regulatory paradigms, including NAMs and QIVIVE [3,175,176].

### 6.1. Organoids: Tissue-Specific and Immune-Competent Models

Human organoids are self-organising, multicellular constructs that recapitulate key aspects of tissue morphology and function. Among the most advanced examples, hepatic organoids derived from pluripotent stem cells have become indispensable tools for the mechanistic evaluation of drug-induced liver injury (DILI) and metabolic safety. These high-fidelity systems reproduce clinical patterns of hepatotoxicity and enable quantitative risk assessment [86,115]. Integrating liver organoids into microfluidic chips further enhances throughput and precision by introducing physiological flow and nutrient exchange [177]. Moreover, multicellular liver constructs designed to model non-alcoholic fatty liver disease (NAFLD) now capture chronic toxicity phenotypes associated with metabolic disorders [178].

Kidney organoids and tubuloids have achieved similar progress, reproducing nephron-like structures, transporter expression profiles, and tubular polarity that enable the study of drug-induced nephrotoxicity and renal clearance. Functional proximal tubule systems allow the investigation of infection, filtration, and injury under near-physiological conditions [179], while quantitative optical imaging enables real-time scoring of renal injury [180]. Comparative analyses highlight their potential as human-relevant alternatives to traditional animal kidney assays [181].

Intestinal organoids provide a complementary model that links absorption, barrier integrity, and microbiota interactions—critical factors in oral pharmacokinetics and first-pass metabolism. Bioengineered intestinal constructs now reproduce epithelial–mesenchymal–neuronal complexity [182], and human enteroid monolayers have been validated as robust and reproducible models of epithelial barrier function and transport [183]. Their application in in vitro–in vivo extrapolation of oral drug disposition has recently been reviewed in detail [184].

Other epithelial organoids extend this approach to barrier tissues such as the cornea, retina, and skin. Retina-on-chip platforms combine retinal organoids with microfluidic circuits to reconstruct neurovascular coupling and photoreceptor–glia interactions [120], while skin organoids have become standardised models for studying irritation, sensitisation, and microbial infection [185,186].

Incorporating immune components within organoid systems adds another layer of complexity. Co-cultures that integrate lymphoid or myeloid cells enable the study of tumour–immune interactions and immunomodulatory toxicity [187,188]. Advanced live cell confocal microscopy now allows continuous, non-invasive observation of these 3D systems, providing real-time visualisation of tissue integrity, morphological adaptation, and cellular viability under both physiological and stress conditions [189,190].

### 6.2. Microfluidics: Organ-on-Chip and Body-on-Chip Systems

MPSs embed human cells within perfused microenvironments that maintain long-term tissue viability, intercellular communication, and physiologically relevant pharmacokinetic gradients. Liver- and kidney-on-chip platforms reproduce key metabolic and excretory functions, generating quantitative endpoints for evaluating DILI and nephrotoxicity [177,191].

Coupled organ circuits—such as gut–liver or liver–kidney configurations—extend this approach by enabling the investigation of metabolite-driven cross-organ effects and systemic clearance [175,192]. Intestinal chips containing self-organising epithelial, mesenchymal, and neuronal components reproduce luminal flow and enteric regulation, providing physiologically coherent models for ADME studies [193].

At the frontier of bioengineering, multi-organ “body-on-chip” and digital-twin systems integrate multiple organ modules within a single circuit to emulate whole-body pharmacokinetics and complex physiological phenomena, including maternal–foetal exchange [194]. Collectively, these innovations represent a decisive shift from static cell culture toward dynamic, systems-level modelling of human biology, offering a powerful bridge between in vitro experimentation and clinical pharmacology.

### 6.3. Three-Dimensional Bioprinting: Standardisation and Reproducibility

3D bioprinting enables the precise, layer-by-layer fabrication of living tissues using bioinks composed of cells and extracellular matrix components. This technology enhances reproducibility, scalability, and architectural fidelity—features that are essential for the regulatory acceptance of organoid-based assays. Extrusion bioprinting has markedly improved the morphological uniformity of kidney constructs [176], while biofabricated hepatic models have demonstrated consistent performance in inter-laboratory toxicity screening studies [195].

Recent technological advances have further expanded the scope of this field. Miniaturised spinning bioreactors now accelerate epithelial organoid production [196], and bioprinted interstitial fibrosis models enable controlled investigation of chronic injury and drug-induced fibrogenesis under defined mechanical conditions [197].

Together, these developments form the foundation for standardised pipelines in 3D tissue fabrication, aligning with Good Cell and Tissue Culture Practice and supporting data harmonisation initiatives that will facilitate broader regulatory adoption.

### 6.4. Translational ADME–Tox Prediction and In Vivo Extrapolation

The convergence of organoid, microfluidic, and bioprinting technologies has ushered in a new era of predictive, mechanism-anchored approaches to human pharmacokinetics and toxicology. When combined with computational modelling and toxicogenomic profiling, these advanced in vitro systems enable robust extrapolation from cellular responses to clinical outcomes.

Recent genomic research has identified polygenic determinants of susceptibility to DILI [198] and mapped molecular response networks through large-scale toxicogenomic studies [199], providing a mechanistic foundation for risk assessment. Liver and intestinal organoid platforms are now routinely employed for ADME profiling and QIVIVE [86,184].

Microphysiological liver systems have demonstrated strong concordance between pharmacokinetic behaviour and dynamic toxicity endpoints [177,200], while multi-organ robotic chips now support automated QIVIVE workflows and integrated data analytics [201]. Collectively, these advances mark a transition from descriptive cytotoxicity testing to predictive, human-centred toxicology grounded in quantitative mechanistic evidence.

Key recommendations for experimental design, model integration, and regulatory alignment are summarised in Box 6.

Box 6 **Practical Guidance for Model Design and Integration.**

- **Combine static and dynamic systems:** Use organoids as foundational tissue modules and integrate them into microfluidic circuits to capture physiological flow, nutrient gradients, and metabolite exchange.
- **Standardise culture conditions:** Define media composition, extracellular matrix parameters, and bioprinting settings to minimise batch variation and improve reproducibility across laboratories.
- **Benchmark with reference compounds:** Validate functional readouts (e.g., albumin, urea, γ-GT, transporter activity) using well-characterised hepatotoxins or nephrotoxins before introducing novel agents.
- **Implement multi-organ connectivity:** Couple intestinal, hepatic, and renal modules to assess systemic ADME and metabolite-driven toxicity, supporting QIVIVE modelling.
- **Integrate computational tools:** Apply PBPK and QIVIVE frameworks to translate microphysiological outputs into clinically relevant exposure predictions.
- **Ensure regulatory alignment:** Follow OECD and FDA recommendations on Good Cell and Tissue Culture Practice and NAMs to support data acceptance and cross-sector harmonisation.

## 7. In Silico Approaches and Computational Toxicology

Computational modelling has become an essential part of modern toxicology. By complementing in vitro systems, it allows prediction of biological effects across large chemical spaces, clarifies underlying mechanisms, and supports quantitative risk assessment [202,203]. The field is increasingly shaped by high-quality, shareable datasets and transparent modelling workflows [204,205] that connect molecular perturbations with physiological responses through pharmacokinetic modelling and quantitative extrapolation frameworks [94,206,207].

**Figure 3:**
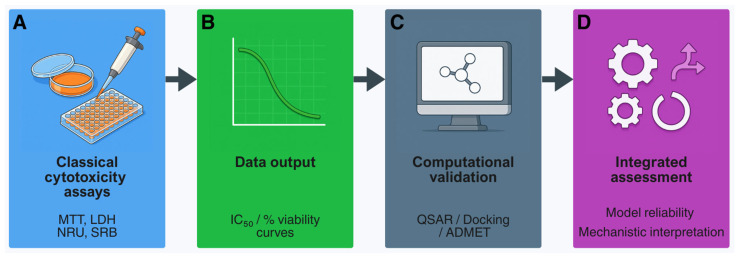
Workflow linking classical cytotoxicity assays with computational analysis. Experimental viability data (IC_50_ or % viability curves) generated from assays such as MTT, LDH, NRU, or SRB feed into QSAR, molecular docking, and ADMET modelling to support mechanistic interpretation and integrated assessment. (**A**) Classical cytotoxicity assays generate baseline viability data using metabolic, membrane-integrity, lysosomal, or biomass-based endpoints. (**B**) Data output includes dose–response curves and derived IC_50_/% viability metrics used as quantitative inputs for modelling. (**C**) Computational validation integrates these data into QSAR, molecular docking, and ADMET workflows to evaluate structural drivers of toxicity and predict biological interactions. (**D**) Integrated assessment combines experimental and in silico evidence to assess model reliability, interpret mechanisms of action, and support decision-making within NAM/IATA frameworks.

The integration of in vitro cytotoxicity data with computational tools is illustrated in Figure 3. Experimental IC_50_ or % viability values obtained from classical assays such as MTT can be used for QSAR, molecular docking (which predicts how a compound interacts with a target protein by simulating ligand–receptor binding), and ADMET analysis to support model validation and mechanistic interpretation [208].

Contemporary in silico toxicology is structured around four methodological pillars:

1. QSAR/read-across, which predicts toxicity directly from chemical structure [205,209];
2. Machine learning (ML) and artificial intelligence (AI), integrating diverse data streams to generate interpretable, multi-endpoint models [202,203,210];
3. PBPK modelling, which simulates ADME to link external exposure with internal dose [206,207]; and
4. QIVIVE, which translates in vitro potency values into human-equivalent exposure metrics [92,93].

The most recent methodological layer (Figure 1E) encompasses computational and integrative frameworks such as QSAR, ML/AI, PBPK, and QIVIVE. All these approaches now operate within harmonised FAIR data and model validation frameworks. However, they share common challenges—particularly uncertainties in metabolic clearance and tissue distribution—that can distort extrapolations if not explicitly tested [207].

An overview of the four computational pillars is provided in Table 2, while a concise, practical workflow from data to regulatory decision making is summarised in Box 7.

Box 7 **Practical workflow for computational toxicology (from data to decision).**

1. **Define the question and endpoint.** Select a suitable modelling family (QSAR or ML) and the kinetic coupling (PBPK or QIVIVE) appropriate to the context.
2. **FAIR data curation.** Standardise identifiers, harmonise units, remove duplicates and outliers, and record provenance and data partitions [204].
3. **Build multiple models.** Compare linear and non-linear learners, define applicability domains, and perform external validation with Y-randomisation checks [202,205].
4. **Interpret and mechanise.** Use structural alerts or feature importance analysis, confirm results by read-across, and document biological plausibility [203,211].
5. **Do the dosimetry.** Convert in vitro concentrations into human-equivalent doses via PBPK/QIVIVE modelling, including uncertainty and sensitivity quantification [92,93,206].
6. **Report transparently.** Publish code, parameters, and domains of applicability with clear caveats for regulatory interpretation [206,207].

### 7.1. Quantitative Structure–Activity Relationships (QSAR), Read-Across, and Cheminformatics

QSAR models mathematically link chemical structure with biological activity or toxicity using statistical and machine learning methods. Structural information is translated into molecular descriptors to infer activity patterns, allowing prediction for untested compounds, prioritisation of further testing, and reduction in animal use under the NAM and IATA frameworks [204,205].

When built from high-quality datasets and applied within a clearly defined domain of applicability, QSAR models can perform reliably for systemic and safety-critical endpoints such as human carcinogenicity or cardiotoxicity [209,212].

Modern guidance stresses adherence to the FAIR principles—findability, accessibility, interoperability, and reusability—to ensure transparency and auditability. Key practical aspects include:

- careful descriptor selection and redundancy control,
- transparent separation of training and validation sets,
- Y-randomisation to exclude chance correlations, and
- explicit uncertainty metrics with confidence bounds [204,205].

Applications range from chronic oral carcinogenicity prediction to cardiac safety screening, where QSAR approaches efficiently flag potential liabilities before costly laboratory testing [209,212]. Comparative analyses consistently show that QSAR delivers the most value when combined with in vitro and preclinical evidence rather than used in isolation [213].

### 7.2. Machine Learning and Artificial Intelligence for Cytotoxicity Prediction

**Figure 4:**
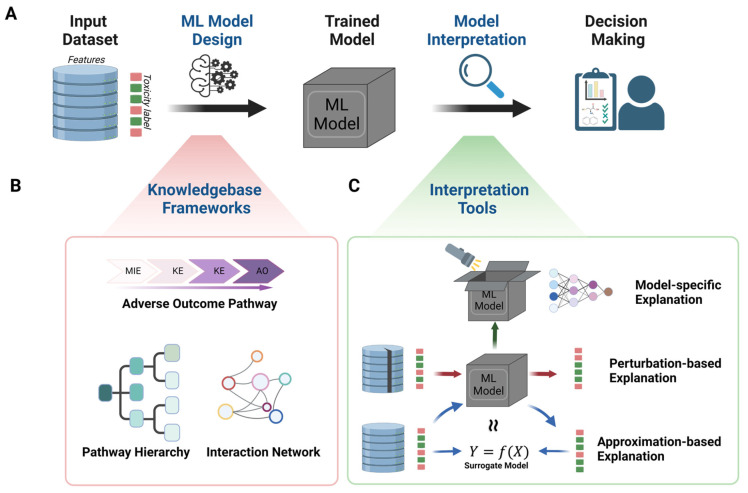
Strategies for developing interpretable machine learning (IML) models for chemical toxicity. (**A**) Pre- and post hoc interpretability strategies applied within ML workflows. (**B**) Knowledge-based frameworks enabling intrinsically interpretable models. (**C**) Application of model interpretation tools for feature analysis and decision support. Reproduced from Jia X., Wang T., Zhu H., *Environ. Sci. Technol.* 2023, 57, 17690–17706 (CC BY 4.0) [203].

Machine learning extends beyond QSAR by capturing non-linear structure–activity relationships and handling complex, multi-endpoint datasets that include omics and imaging features. Publicly accessible platforms such as ProTox 3.0 now provide continuously updated toxicity models with user-friendly interfaces suitable for both academia and industry [210]. An overview of strategies for developing interpretable machine learning models in chemical toxicity is shown in Figure 4.

Interpretability remains central to regulatory acceptance. Mechanism-aware and model-agnostic techniques—such as feature importance mapping and attention-based visualisation—allow predictions to be linked to specific chemical substructures or biological pathways, thereby increasing confidence in model outputs [203].

Guidelines from recent studies clarify when to favour classical learners (e.g., random forests) versus deep neural networks and emphasise the importance of rigorous external validation to prevent overfitting and ensure reproducibility across datasets [202].

End-to-end ML pipelines now automate data curation, descriptor generation, training, and deployment, reducing manual bias and improving reproducibility [214]. Explainable ML models for dermal toxicity achieve accuracy comparable to traditional baselines while providing clear insight into mechanistic drivers [215]. In cardiac safety assessment, curated datasets of hERG (human Ether-à-go-go-Related Gene) channel inhibition highlight that model reliability depends heavily on data quality, curation of negatives, and threshold optimisation [216].

Overall, these advances show that ML delivers greatest value when trained on well-annotated, purpose-specific datasets, implemented through reproducible pipelines, and accompanied by interpretable outputs. This union of transparency and mechanistic insight has moved ML from an exploratory tool to a credible, fit-for-purpose element of regulatory toxicology [214,215,216].

### 7.3. Physiologically Based Pharmacokinetic (PBPK) Modelling

PBPK models quantitatively describe how chemicals are absorbed, distributed, metabolised, and excreted by representing human physiology—blood flow, tissue partitioning, and metabolism—in a mechanistic framework. They link in vitro activity thresholds to predicted tissue concentrations in human populations, accounting for variability due to age, comorbidities, and drug–drug interactions (DDI) [206].

From both industrial and regulatory perspectives, three key pillars define robust PBPK practice:

1. Population relevance: evaluation of specific subgroups such as paediatrics, pregnancy, or hepatic/renal impairment.
2. Uncertainty management: systematic sensitivity analysis of physiological and chemical parameters to assess influence on predictions.
3. Model qualification: benchmarking against reliable clinical reference data [206].

Within model-informed drug development (MIDD), PBPK supports first-in-human dosing, DDI prediction, and extrapolation to sensitive populations where direct data are limited [94]. Recent extensions include models accounting for obesity-related changes in organ perfusion and clearance [217] and AI-assisted PBPK frameworks for nanoparticle pharmacokinetics [218]. Specialised modules have been introduced for nanoparticle dynamics—addressing protein corona formation and mononuclear phagocyte uptake [219].

Importantly, reproducibility requires external validation. Multi-centre comparisons, such as PBPK qualification in pregnancy, demonstrate that harmonised workflows can yield reliable maternal–foetal exposure predictions [207].

### 7.4. Quantitative In Vitro–In Vivo Extrapolation (QIVIVE)

**Figure 5:**
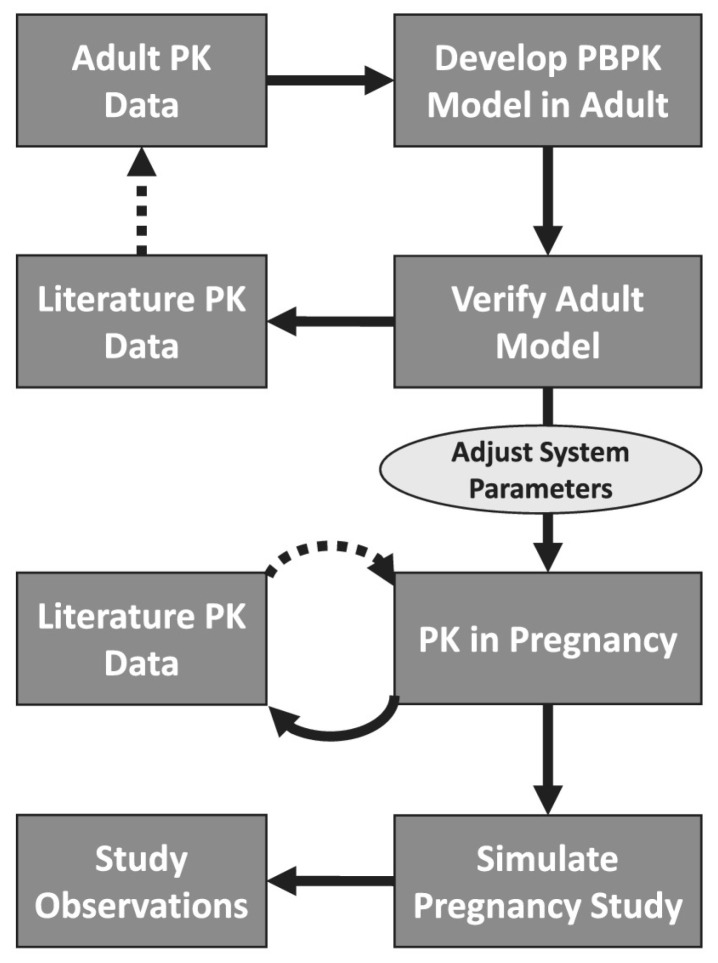
Physiologically based pharmacokinetic (PBPK) modelling in pregnancy—model validation and reproducibility. Solid arrows denote the main modelling workflow, while dashed arrows represent supplementary literature-derived inputs used to support model development and verification. Reproduced from Silva L.L. et al., *Br. J. Clin. Pharmacol.* 2022, 88, 1441–1451. © John Wiley & Sons [207]. Reproduced with permission. Licence No. 6143080459988.

QIVIVE integrates in vitro concentration–response data with PBPK or physiologically based toxicokinetic (PBTK) models to estimate human-relevant exposure levels [92,93]. A representative example of PBPK model validation in a special population (pregnancy) is shown in Figure 5.

The process typically involves four key elements:

(i) correction of in vitro concentrations for plastic and protein binding;
(ii) determination of binding fractions in blood and tissues;
(iii) measurement of metabolic and excretory clearance; and
(iv) definition of the relevant exposure metric—Cmax, AUC, or steady state—with quantified uncertainty.

High-throughput PBTK workflows now enable QIVIVE for thousands of compounds, integrating internal dose predictions with bioactivity profiles for screening-level risk ranking [93]. Mechanistic key events can be embedded directly within the PBPK–QIVIVE chain—for instance, using epigenetic markers to refine risk estimates for polyfluoroalkyl substances (PFAS) [185], or achieving cross-species concordance for acetylcholinesterase inhibition when kinetic parameters are well characterised [220].

Proper diagnostics and uncertainty analyses are essential: inaccurate assumptions regarding clearance or partitioning can distort outcomes, so sensitivity and probabilistic error propagation should always be conducted [207]. Beyond single viability endpoints, phenotypic profiling—for example, the Cell Painting assay offers mechanistic fingerprints to anchor the in vitro point of departure and strengthen translational validity [66].

In practice, reproducible QIVIVE requires simulation of tissue concentration–time curves within a PBPK framework, selection of the appropriate exposure metric, and execution of global or Monte Carlo uncertainty analyses, all implemented in transparent, script-based pipelines for traceability [92,93]. Properly applied, QIVIVE transforms in vitro findings into quantitative human risk estimates, positioning in silico toxicology as an integrative partner—rather than a replacement—for experimental systems.

Key references, practical guidance, and step-by-step implementation details are provided in Box 8 and Box 9.

Box 8 **Key resources for QIVIVE and PBPK modelling.**

**Reviews and Methods**

- Practical roadmaps for QIVIVE and integration into IATA [92]
- High-throughput PBTK for QIVIVE at scale [93]
- PBPK for decision making and uncertainty analysis [206]
- Model-informed development for special populations [94]
- Diagnostics for QIVIVE mis-specification [206,207]
- Linking phenotypic profiling with QIVIVE (Cell Painting) [66]

**Case Studies**

- PFAS: epigenetic key event integration within PBPK [221]
- AChE inhibition: kinetic cross-species concordance [220]

**How-To Sources**

- Open-source tools, example datasets, and regulatory guidelines for QIVIVE implementation [92,206,222]

Box 9 **Practical workflow for QIVIVE and PBPK implementation.**

**(i)**  **Free (unbound) assay concentration**. Correct for plastic and protein binding to avoid overestimation [92,222].
**(ii)**  **Binding in blood and tissues.** Include unbound plasma and tissue fractions; adjust blood-to-plasma ratios; apply partitioning models for realistic distribution [92,206].
**(iii)**  **Clearance via metabolism and transport/excretion** Determine intrinsic clearance (Clint) using human hepatocytes or microsomes, scale appropriately, and include transporter-mediated processes validated through sensitivity analysis [93,206,223].
**(iv)**  **Exposure metric and uncertainty.** Select *C*max, AUC, or steady-state concentration; report associated uncertainty and perform sensitivity checks before using outputs for decision making [92,206].

### 7.5. Software and Web-Based Tools for In Silico Toxicity and Pharmacokinetic Modelling

The increasing sophistication of computational toxicology has been driven by the emergence of specialised software and web-based tools that integrate chemical structure, biological response, and pharmacokinetic behaviour. These platforms are now indispensable for predicting cytotoxicity, absorption, distribution, metabolism, and excretion profiles before experimental testing. When combined with experimental validation, they support more predictive, mechanistically grounded approaches to safety assessment.

#### 7.5.1. QSAR and Machine Learning Platforms

Tools such as the OECD QSAR Toolbox, ProTox 3.0 [210], and ComptoxAI [214] are among the most frequently used resources for chemical hazard prediction. They enable rapid screening across large chemical libraries, highlighting potential toxicophores and mechanistic alerts. Their main strengths include transparency, accessibility, and standardised model documentation. However, their predictive reliability depends heavily on the quality and diversity of training data, while deep learning frameworks, although powerful, can lack interpretability and transferability.

#### 7.5.2. PBPK and ADME–Tox Modelling Suites

Mechanistic pharmacokinetic simulations are routinely performed using Simcyp Simulator, GastroPlus, and the open source PK-Sim/MoBi environment [206]. These tools quantitatively describe tissue distribution and metabolic clearance, enabling QIVIVE and virtual population studies. Their advantages lie in the integration of physiological realism and inter-individual variability; nonetheless, they require accurate physicochemical input data and careful uncertainty analysis to avoid misleading exposure predictions.

Recent work by Hassan et al. [224] demonstrated how molecular docking, ADME prediction, and PBPK modelling can be combined to investigate the pharmacokinetic behaviour of natural compounds. This study illustrates how in silico pipelines can complement in vitro and in vivo experimentation, improving translational confidence in toxicity and efficacy evaluation.

A concise comparison of representative software and web-based tools used for in silico cytotoxicity and pharmacokinetic prediction is presented in Table 3.

Collectively, these computational resources accelerate early-stage screening, reduce experimental burden, and foster reproducibility through transparent digital workflows. Yet, their use should always be coupled with biological validation and sensitivity analysis. The integration of QSAR, machine learning, and PBPK tools with advanced in vitro systems now represents the most effective route towards quantitative, mechanistically anchored, and human-relevant toxicology.

## 8. Integrated Approaches and Regulatory Perspectives

The global transition from descriptive, animal-based testing to predictive and mechanistically anchored toxicology has been propelled by the emergence of IATA and NAMs. These frameworks weave together in vitro, in silico, and in chemico data with existing knowledge to support regulatory decisions aligned with the 3Rs principle [6].

Rather than denoting single assays, IATA represent structured, evidence-based strategies that combine data from validated experimental systems, computational models, and expert judgement to establish hazard or potency. Their architecture is deliberately flexible and transparent, designed to be fit-for-purpose and adaptable across different chemical domains and regulatory settings [225].

### 8.1. From Concept to Practice: Building Confidence in NAMs

Scientific and regulatory confidence in NAMs has grown steadily over the past decade under the coordinated guidance of organisations such as the OECD, EURL ECVAM (EU Reference Laboratory for Alternatives to Animal Testing), and U.S. agencies including the Environmental Protection Agency (EPA), FDA, and NIH. Consensus has emerged that the credibility of NAMs does not rely on a one-to-one substitution of animal studies but rather on the demonstration of reproducibility, mechanistic coherence, and well-defined domains of applicability [226].

NAMs now span a broad technological spectrum—from high-content imaging and transcriptomic profiling to organ-on-chip microphysiological systems and computational modelling. Their regulatory acceptance is evaluated through context-of-use validation, whereby a method’s reliability is assessed within the specific decision framework for which the data are intended [91,227].

**Figure 6:**
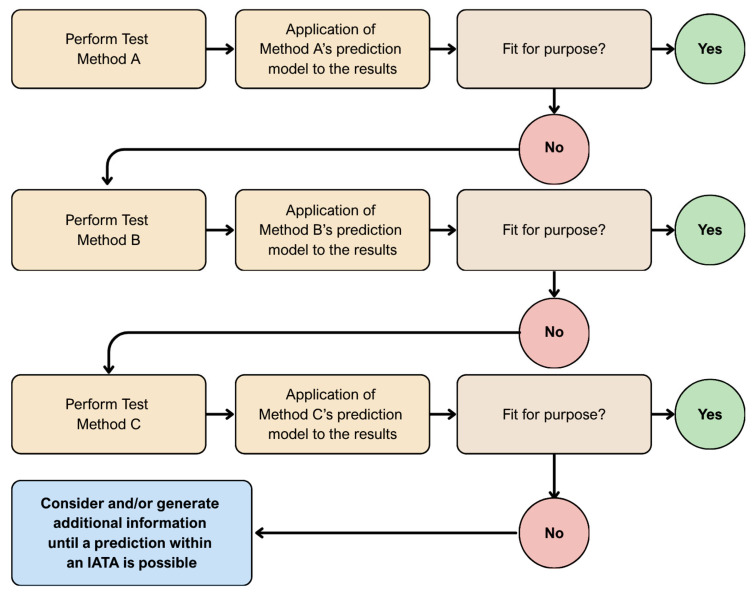
Sequential Testing Strategy (STS) within the IATA framework. Each method (A–C) generates a prediction that is evaluated for regulatory fit-for-purpose; a “Yes” outcome terminates the sequence, whereas “No” triggers the next testing tier or the need for additional information. Adapted from OECD Guidance Document No. 255 (2017) [228].

Within the IATA paradigm, the Sequential Testing Strategy (STS) provides a generic decision–logic framework that structures how individual methods are applied. STS does not prescribe which assays must be used; instead, it defines *how* evidence is generated, evaluated, and integrated in a transparent and stepwise manner. At each tier, the outcome of a test is assessed for regulatory ‘fit-for-purpose’: if the prediction is sufficiently reliable for the intended decision context, testing can stop. If not, additional or higher-tier information is required. This tiered logic minimises unnecessary experimentation, aligns testing with mechanistic understanding, and ensures that data streams of varying complexity are incorporated in a scientifically coherent way [228]. An OECD generic version of this structure is shown in Figure 6.

Selected examples of this principle are summarised in Box 10, highlighting validated NAMs accepted within defined regulatory contexts.

Box 10 **Context-of-use validation: how NAMs gain regulatory credibility.**

Regulatory confidence in NAMs is achieved through context-of-use validation, which establishes a method’s reliability for a defined regulatory purpose rather than as a universal replacement for animal testing.

**Examples:**

- Skin sensitization—The DA (OECD TG 497) is validated for identifying sensitising chemicals but not for potency ranking or quantitative risk assessment [229].
- Skin irritation—Reconstructed human epidermis models (OECD TG 439) are accepted for classification and labelling but not for chronic or systemic toxicity testing [230].
- Microphysiological liver models—Evaluated by the U.S. FDA for detecting drug-induced liver injury in preclinical settings, though not yet validated for whole-body toxicity prediction [226,227].

**Key principle:**

Confidence in a NAM depends on demonstrated reliability within its regulatory context—each method is accepted only for what it has been proven to do.

Collaborative initiatives such as APCRA (Accelerating the Pace of Chemical Risk Assessment) and PARC (Partnership for the Assessment of Risks from Chemicals) have further strengthened harmonisation through shared case studies and alignment of interpretive criteria [231]. More recently, expert groups have called for unified validation principles and standardised reporting to support cross-jurisdictional acceptance of NAM-derived data [232]. Ultimately, successful implementation depends not only on technological maturity but also on the preparedness of the regulatory ecosystem—its training, resources, and institutional culture—which determine how effectively innovation is embedded in practice [233].

### 8.2. Case Studies and Regulatory Uptake

The OECD introduced the concept of IATA as a flexible framework that combines in silico, in chemico and in vitro data with existing knowledge to support hazard identification and risk assessment [228]. Within this framework, the STS defines a structured, stepwise process in which individual methods are applied in sequence and each prediction is assessed for regulatory fit-for-purpose. As outlined in Section 8.1 (Figure 6), this generic STS provides the conceptual basis for tiered testing, reducing unnecessary experimentation while ensuring transparent and reliable decision making.

Validated IATA and DAs have progressed from theoretical constructs to operational tools within several OECD Test Guidelines, providing practical alternatives to animal testing.

In the field of skin corrosion and irritation, reconstructed human epidermis models such as EpiDerm™, SkinEthic™, and epiCS—when combined with in chemico assays like the Direct Peptide Reactivity Assay (DPRA)—are formally recognised under OECD TG 431 and TG 439, marking a mature application of IATA principles in regulatory toxicology [230,234,235]. Similarly, eye irritation testing has been revolutionised by in vitro corneal models such as EpiOcular™ and SkinEthic™ HCE, which underpin OECD TG 492 and effectively replace the classical Draize rabbit eye test [35,236,237,238]. Real-time impedance-based monitoring now enhances these models, allowing quantitative distinction between reversible and irreversible injury.

Advances are also evident in developmental and reproductive toxicity—traditionally a major barrier to non-animal assessment. Modern IATA integrate human pluripotent stem cell differentiation assays with multi-omics and PBPK/QIVIVE modelling to connect early mechanistic perturbations with adverse developmental outcomes [239,240].

New-generation assay platforms such as ReproTracker, PluriLum, and the UKN4DNT framework now make it possible to assess embryotoxicity in a structured and mechanistically informed way. By analysing transcriptomic and proteomic patterns, these systems reveal how toxicants disturb key developmental processes—including neurogenesis, cardiogenesis, and morphogenesis—within human stem cell-based models (see Box 11).

Box 11 **Emerging Platforms for Developmental and Reproductive Toxicity Testing.**

- **ReproTracker**—Tracks differentiation of human pluripotent stem cells into germ layers to detect embryotoxic and teratogenic effects through gene expression markers [239].
- **PluriLum Test**—Combines stem cell differentiation with high-content imaging and transcriptomics, generating mechanistic fingerprints of disrupted morphogenesis [240].
- **UKN4DNT Framework**—Integrates neural differentiation and omics-level profiling to identify key events in developmental neurotoxicity pathways [239,240].
- **PBPK/QIVIVE Coupling**—Translates in vitro concentration–response data into human-equivalent exposure levels for quantitative risk assessment [220,221].

When linked with pharmacokinetic modelling, these data support quantitative points of departure and safety margins without recourse to animal studies [92,93,241,242]. A notable example of next-generation risk assessment (NGRA) is the daidzein read-across case, in which exposure modelling, in vitro assays, and computational predictions were integrated into a tiered workflow yielding regulatory-quality safety conclusions [243]. Collectively, these examples illustrate how IATA and NAMs are transforming mechanistic concepts into practical regulatory instruments.

**Figure 7:**
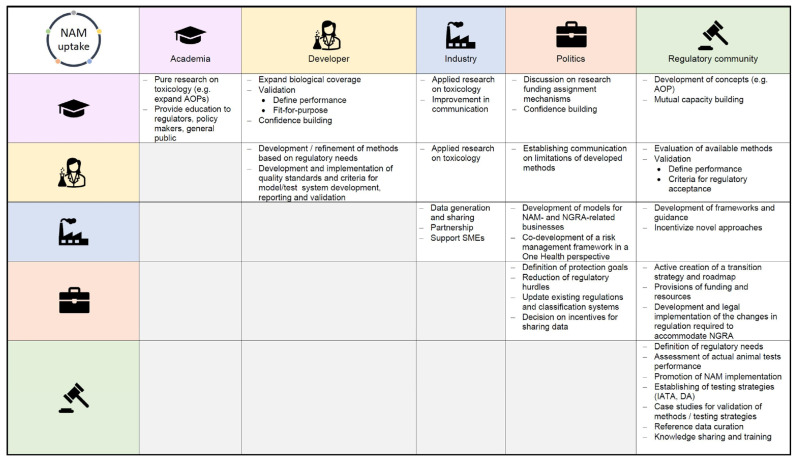
Integration of New Approach Methodologies (NAMs) and IATA frameworks in regulatory toxicology. Key responsibilities of academia, regulatory agencies, and industry in transitioning from animal-based testing to NAM-grounded evaluation. Reprinted from Schmeisser et al., *Environ. Int.* 2023, 178, 108082 [91] (CC BY 4.0).

The system-wide implementation of New Approach Methodologies (NAMs) and Integrated Approaches to Testing and Assessment (IATA) depends not only on the performance of individual methods but also on effective collaboration between academia, industry, and regulatory bodies. Recent initiatives emphasise that the transition towards non-animal, mechanism-based testing should focus not on whether NAMs should be used, but how and when they can be integrated into existing regulatory frameworks. Figure 7 summarises the key responsibilities and interaction points of major stakeholders in this process, highlighting that regulatory transformation relies on data harmonisation, cross-sector validation, standardised protocols, and sustained capacity building across scientific and institutional domains [91].

### 8.3. Global Regulatory Perspectives

The OECD has formalised principles underpinning IATA and NAMs in its Guidance Document No. 255 on the reporting of DAs (2017) [228] and Guidance Document No. 311 on Weight-of-Evidence evaluation (2019) [244], which together establish standards for data integration, uncertainty analysis, and transparent reporting. These documents support the DA concept exemplified in OECD TG 497 on skin sensitisation [229].

Parallel developments at the FDA and EMA demonstrate similar intent. The FDA’s Roadmap to Reducing Animal Testing (2025) encourages inclusion of NAM-generated data in preclinical submissions [241], while the EMA’s Horizon Scanning Report on NAMs (2025) highlights their centrality to future regulatory science [242]. Within the EU, EURL ECVAM continues to track progress through its annual Status Reports [245,246,247], ensuring transparency in validation and acceptance. Notably, recent OECD updates introduced an IATA for Phototoxicity [248] and revised Test Guideline 442D to include EpiSensA [249], extending non-animal strategies for skin sensitisation.

Although regional differences persist in validation criteria and reviewer expertise, these collective actions demonstrate a strong and coordinated global momentum toward harmonised, mechanistically grounded regulation.

Representative examples of IATA/NAM implementation across regulatory endpoints are shown in Table 4.

### 8.4. Outlook and Emerging Trends

Looking ahead, the convergence of AI, multi-omics, and MPS networks is poised to enhance the predictive and translational scope of NAMs. Rapid progress in QIVIVE is enabling quantitative linkage between in vitro dose–response relationships and human exposure scenarios [92,220]. In parallel, computational innovations—such as machine learning-optimised PBPK models and genetic algorithm parameter estimation—are refining model representativeness and reproducibility [218,250].

On the policy side, the CHANGE Initiative (2024–2026) (Collaboration to Harmonize the Assessment of Next Generation Evidence) seeks to accelerate NAM adoption through coordinated action across governance, funding, and education [233]. Harmonised IATA and DAs are now increasingly acknowledged as formal decision making frameworks across regulatory systems [91]. Beyond human health, new IATA/NAM applications are emerging in ecotoxicology and mixture assessment, extending mechanistic and ethical principles to environmental safety [251,252].

Together, these developments demonstrate that IATA and NAMs now form the operational backbone of 21st century toxicology—uniting computational modelling, AI-assisted data interpretation, and human-relevant biology into a predictive, quantitative, and ethically sustainable science.

## 9. Limitations and Future Perspectives

Although cytotoxicity testing has advanced substantially, important limitations remain across classical, mechanistic, and human-relevant approaches. Classical colorimetric assays such as MTT, LDH, and NRU laid the foundation of in vitro toxicology [8,10,11], but their simplicity prevents them from capturing complex toxic mechanisms and systemic responses [4]. These assays are also prone to optical or chemical interference and may overlook early or adaptive cellular changes.

Modern high-throughput and high-content screening technologies greatly expanded the ability to map cellular perturbations [16], yet the resulting datasets require sophisticated analysis, appropriate viability counterscreens, and robust quality control to reduce false mechanistic interpretations [17,18], but still face challenges such as incomplete maturation, inter-line variability, and ethical constraints in the case of hESCs.

Three-dimensional organoids, organ-on-chip devices, and bioprinted tissues recreate physiological microenvironments and tissue–tissue interactions [22,24], yet broader implementation is hindered by technical complexity, cost, and limited inter-laboratory reproducibility. Similarly, integrative computational approaches—including QSAR, ML/AI, PBPK, and QIVIVE—depend on high-quality datasets, accurate kinetic parameters, and transparent uncertainty analysis [29,30].

Looking ahead, deeper integration across experimental and computational layers will likely shape the next phase of development. AI-assisted models combined with omics signatures and QIVIVE may enable multiscale prediction of toxic responses [203]. Multi-organ microphysiological systems, including emerging body-on-chip configurations [27], are expected to support dynamic evaluation of systemic toxicity and inter-organ communication. Patient-specific hiPSC-derived platforms offer a path toward precision toxicology by capturing inter-individual variability in chemical susceptibility [17].

Together, these directions highlight both the current methodological gaps and the opportunities for creating predictive, mechanistically grounded cytotoxicity frameworks.

## 10. Conclusions

Cytotoxicity testing has undergone a substantial transformation, progressing from basic viability assays toward multifaceted, mechanistically informed systems that support both biological interpretation and regulatory decision making. Rather than relying on isolated endpoints, contemporary in vitro toxicology increasingly integrates biochemical, morphological, kinetic, and computational evidence to generate coherent and biologically plausible toxicity profiles. This shift reflects a broader movement within the field toward methods that enhance human relevance, reproducibility, and translational value [2,3].

Across biomedical applications, these methodological advances have strengthened early-phase safety evaluation by enabling more reliable detection of subtle, adaptive, and mechanistically anchored cellular responses [1]. In regulatory science, they form the conceptual basis for NAMs and IATA, which aim to reduce animal use while improving mechanistic transparency and decision confidence [6]. Human-derived models, multi-parametric imaging, and kinetic assays now operate alongside computational tools to create evidence frameworks that align with current expectations for robustness, traceability, and biological interpretability.

As experimental and computational approaches continue to converge, cytotoxicity testing is becoming an increasingly predictive and integrative discipline—one capable of linking molecular perturbations with cellular and systemic responses within coherent, mechanistically grounded evaluation frameworks. This evolution supports safer drug development, more sustainable chemical innovation, and more human-relevant regulatory assessments. A concise overview of the historical milestones and emerging methodological directions is provided in Table 5.

## Figures and Tables

**Table 1:** Comparison of hESC-, hiPSC-, and adult stem cell–based models in cytotoxicity testing.

Feature	hESCs	hiPSCs	Adult Stem Cells (HSCs, MSCs)
Source	Inner cell mass of human blastocysts (IVF surplus embryos)	Reprogrammed adult somatic cells (fibroblasts, blood, urine) using Yamanaka factors	Bone marrow, peripheral blood (HSCs), adipose or umbilical cord tissue (MSCs)
Potency	Pluripotent (all germ layers)	Pluripotent (patient-specific, variable)	Multipotent (restricted to specific tissue lineages)
Applications	Developmental toxicity; cardiac, hepatic, neuronal, epithelial, ocular models [101,107]	Cardiotoxicity, hepatotoxicity, developmental neurotoxicity, renal and ocular assays, precision toxicology [113,116,118]	Immunotoxicity, myelotoxicity, biomaterial and nanomaterial cytotoxicity [144,145,147,149]
Advantages	Natural pluripotency; reproducible protocols; validated differentiation	Ethically acceptable; scalable; patient-specific	Easy access; ethically uncontroversial; tissue-relevant
Limitations	Ethical controversy; limited access; teratoma risk	Variability; incomplete maturation; donor heterogeneity	Limited potency; donor variability; senescence
Ethical/Legal	Strict oversight (NIH Registry, EU Directive 2004/23/EC, ISSCR)	Informed consent; data protection (ISSCR 2021)	Standard medical consent; minimal restrictions

**Table 2:** Summary of in silico pillars for cytotoxicity/health endpoints.

Method	Primary Inputs	Typical Outputs	Strengths	Common Pitfalls	Use Cases	Key Refs
QSAR/ read-across	Molecular structures, curated labels	Class or continuous risk	Fast, interpretable	Limited domain, data leakage	Early hazard identification	[204,205,209]
ML/AI	Structures + omics/phenotypes	Multi-endpoint predictions	Handles non-linear, multi-task data	Interpretability drift	Portfolio triage, prioritisation	[202,203,210]
PBPK	Physiology, ADME parameters	Tissue concentration–time (C(t))	Human- relevance	Parameter uncertainty	Populations, drug–drug interaction (DDI), exposure assessment	[94,206,207]
QIVIVE	In vitro ECx + PBPK	Human-equivalent dose	Translational, mechanistic	Mis-specified clearance	Screening-level risk, potency estimation	[92,93]

**Table 3:** Overview of commonly used in silico tools for toxicity and pharmacokinetic prediction.

Tool/Platform	Main Function	Key Advantages	Typical Limitations	Ref
OECD QSAR Toolbox	Structure-based prediction, read-across	Open-access, regulatory credibility, mechanistic alerts	Limited chemical domain; manual curation required	[204]
ProTox 3.0	Web-based toxicity prediction (ML/QSAR hybrid)	Intuitive interface; wide coverage of endpoints	Dependent on curated training data; black box algorithms	[210]
ComptoxAI	AI-assisted chemical hazard modelling	Automated data handling; reproducible pipelines	Model transparency and interpretability challenges	[214]
Simcyp Simulator	PBPK/PK–PD modelling, virtual clinical trials	Population variability, organ impairment modules	Requires licenced software; parameter sensitivity	[206]
GastroPlus	PBPK-based oral absorption and systemic PK	Physiological realism, QIVIVE capability	Cost, complex calibration	[94]
PK-Sim/MoBi	Open-source PBPK modelling suite	Transparency; flexible scripting; reproducible models	Requires expert parameterisation	[207]
SwissADME	Free web ADME/Tox and drug likeness predictor	Easy access, visual output, rapid screening	Limited mechanistic depth; qualitative outputs	[205]

**Table 4:** Examples of IATA/NAM implementation across regulatory endpoints.

Endpoint	Primary NAM(s)/DA	OECD TG/Guidance	Regulatory Scope	Status/ Notes	Key Refs
Skin sensitisation	DPRA + KeratinoSens™ + h-CLAT (DA)	OECD TG 497 (2025)	Classification and labelling	Fully accepted	[226,229]
Skin irritation	Reconstructed epidermis (EpiDerm™, SkinEthic™, epiCS)	OECD TG 439 (2025)	Classification and labelling	Fully accepted	[225,230]
Eye irritation	Reconstructed corneal epithelium (EpiOcular™, SkinEthic™ HCE)	OECD TG 492 (2025)	Classification and labelling	Accepted; replaces Draize test	[236,237,238]
Phototoxicity	IATA for Phototoxicity	OECD Guidance No. 397 (2024)	Screening/ Hazard ID	Recently introduced	[248]
Nanomaterial inhalation	Grouping/ Read-Across Approach	–	Occupational risk assessment	Emerging application	[235]
Developmental toxicity	PluriLum/ReproTracker + PBPK/QIVIVE	–	Developmental and Reproductive	Under validation	[239,240]

**Table 5:** Milestones and emerging directions in cytotoxicity testing.

Stage/Era	Key Advances	Representative Methods/Systems	Main Impact
Classical (1980s–2000s)	Colorimetric and metabolic viability assays	MTT, LDH, Neutral Red, Resazurin	Foundation of in vitro toxicology; standardised endpoints; regulatory benchmarks
Mechanistic (2000s–2010s)	High-throughput and high-content screening; mechanistic readouts	HCI, Cell Painting, flow cytometry, xCELLigence	Multiparametric mechanistic insight; reduction in false positives/negatives
Human-relevant (2010s–2020s)	Stem cell–based and 3D models	hPSC/hiPSC assays, organoids, organ-on-chip	Human-specific predictive systems; translation to tissue- and organ-level toxicity
Computational and Integrative (2020s–present)	AI, PBPK/QIVIVE, NAMs/IATA frameworks	Machine learning, QIVIVE, body-on-chip	Mechanistic–quantitative risk assessment; regulatory adoption of non-animal evidence
Emerging (Future)	Personalised, multi-organ, and AI-driven toxicology	Patient-derived hiPSC models, multi-MPS networks, digital twins	Predictive, individualised safety assessment; convergence of toxicology and precision medicine

## Data Availability

No new data were created or analysed in this study. Data sharing is not applicable to this article.

## Abbreviations

The following abbreviations are used in this manuscript:

2D/3D	Two-/Three-Dimensional Cell Culture
3Rs	Replacement, Reduction, and Refinement
ADME	Absorption, Distribution, Metabolism, and Excretion
AI	Artificial Intelligence
CFU	Colony-Forming Unit
DA	Defined Approach
DDI	Drug–drug interaction
DILI	Drug-Induced Liver Injury
EMA	European Medicines Agency
FDA	Food and Drug Administration
HCI	High-Content Imaging
hESC	Human Embryonic Stem Cell
hiPSC/iPSC	(Human) Induced Pluripotent Stem Cell
hiPSC-CM	hiPSC-Derived Cardiomyocyte
HSC	Hematopoietic Stem Cell
qHTS	Quantitative High-Throughput Screening
IATA	Integrated Approaches to Testing and Assessment
ISSCR	International Society for Stem Cell Research
LDH	Lactate Dehydrogenase
ML	Machine Learning
MPS	Microphysiological System
MTT	3-(4,5-Dimethylthiazol-2-yl)-2,5-diphenyltetrazolium bromide
NAMs	New Approach Methodologies
NIH	National Institutes of Health
NRU	Neutral Red Uptake
OECD	Organisation for Economic Co-operation and Development
PBPK	Physiologically Based Pharmacokinetic (Modelling)
PFAS	Polyfluoroalkyl Substances
PI	Propidium Iodide
PSC	Pluripotent Stem Cell
QIVIVE	Quantitative In Vitro–In Vivo Extrapolation
QSAR	Quantitative Structure–Activity Relationship
ROS	Reactive Oxygen Species
RTCA	Real-Time Cell Analysis
SRB	Sulforhodamine B
STS	Sequential Testing Strategy
tcpl	ToxCast Pipeline for Curve Fitting
Tox21/ToxCast	U.S. Toxicology Data Programs for High-Throughput Screening
